# Thank you to all our manuscript reviewers in 2015

**DOI:** 10.1186/s13053-016-0047-4

**Published:** 2016-02-29

**Authors:** Jan Lubinski, Rodney J. Scott, Rolf Sijmons, Sarah M. Theissen

**Affiliations:** Department of Genetics and Pathomorphology, Pomeranian Medical University, Szczecin, Poland; Discipline of Medical Genetics, Faculty of Health, University of Newcastle and Hunter Medical Research Institute, New South Wales, Australia; Department of Genetics, University Medical Center Groningen, Groningen, The Netherlands; BioMed Central, 236 Gray’s Inn Road, London, WC1X 8HB UK

## Abstract

The editors of *Hereditary Cancer in Clinical Practice* would like to thank all our reviewers who have contributed to the journal in 2015.

Without the participation of skilful reviewers, no academic journal could succeed, and we are grateful to the committed individuals who have given their time and expertise to the peer review of manuscripts for *Hereditary Cancer in Clinical Practice*. We look forward to your continued support in 2016.

**Stefan Aretz**

Germany

**Gabriela Balogh**

Argentina

**Elizabeth Bancroft**

UK

**Morgan Butrick**

USA

**Berta Campos**

Spain

**John V Conaglen**

New Zealand

**Cezary Cybulski**

Poland

**Tadeusz Debniak**

Poland

**Pawel Domagala**

Poland

**Thilo Dork**

Germany

**Dagmara Dymerska**

Poland

**D Gareth Evans**

UK

**Thomas Hansen**

Denmark

**Shirley Hodgson**

UK

**Frans Hogervorst**

Netherlands

**Evgeny Imyanitov**

Russia

**Arvids Irmejs**

Latvia

**Anna Jakubowska**

Poland

**Wojciech Kluźniak**

Poland

**Kristina Lagerstedt Robinson**

Sweden

**Marcin Lener**

Poland

**Annika Lindblom**

Sweden

**Finlay Macrae**

Australia

**Diptasri Mandal**

USA

**Alexandra Martins**

France

**Cliff Meldrum**

Australia

**Janusz Menkiszak**

Poland

**Caterina Mian**

Italy

**Pal Moller**

Norway

**Steven Narod**

Canada

**Jan Oosterwijk**

Netherlands

**Carmen Radecki Breitkopf**

USA

**Malgorzata Stawicka Nielacna**

Poland

**Bente Talseth-Palmer**

Australia

**Amanda Toland**

USA

**Annemieke van der Hout**

Netherlands

**Rob van der Luijt**

Netherlands

**Michelle Wong-Brown**

Australia

**Xingnan Zheng**

USA

